# Experiences of teachers and students on school closures and its consequences during the COVID-19 pandemic in Nyarugusu refugee camp, Tanzania

**DOI:** 10.1371/journal.pgph.0002917

**Published:** 2024-03-18

**Authors:** Elizabeth H. Shayo, Godfrey Mubyazi, Vivien Barongo, Mtumwa Bakari, Zenais Kiwale, Camilla Fabbri, Ellen Turner, Katherine Rodrigues, Karen Devries

**Affiliations:** 1 National Institute for Medical Research, Barack Obama Drive, Dar-es-salaam, Tanzania; 2 London School of Hygiene and Tropical Medicine, London, United Kingdom; 3 International Rescue Committee, New York, New York, United States of America; Royal Holloway University of London, UNITED KINGDOM

## Abstract

Tanzania has experienced several waves of COVID-19 since it was first detected in the country. During the first wave, Tanzania took several measures to prevent wider virus transmission with school closures being one of them. All areas and institutions were targeted, including the refugee camps in Kigoma region. Despite the abundant evidence generated in relation to the effects of the pandemic and associated school closures globally, there has been a paucity of literature exploring the experiences of teachers and students in humanitarian settings. We conducted a qualitative study to explore COVID-19 related school closures in Nyarugusu refugee camp. We aimed to describe teachers’ and students’ experiences and perceived consequences of school closures. In-depth interviews with teachers and students were conducted in September 2020 in Burundian and Congolese schools in the context of a cluster randomised trial of EmpaTeach, a school-based violence prevention intervention. A total of 44 individuals (29 teachers and 15 students) were interviewed. A phenomenological theoretical framework was used to guide the content analysis. Findings indicated that the COVID-19 pandemic was generally seen as frightening by refugees. Study participants understood the importance of school closures to prevent transmission of the virus, but various negative consequences were reported by both teachers and students. These included perceived mental health difficulties such as stress, depression and anxiety associated with the worry of infection, idleness, and disruption of education. Participants also perceived an increase in occurrences of early marriages and unplanned pregnancies, which they thought contributed to increased school dropout. Participants identified the main causes of such outcomes as a lack of parental supervision, children’s lack of restraint and poor character, and a lack of alternative teaching practices (such as online or remote learning) to keep the students busy while at home. Children were held accountable for their faults with little support from the adults. Our findings suggest that there is an urgent need to strengthen child protection programming to support children and their communities during emergencies and provides protective environments such as school and education. There is a critical need to develop preparedness plans for future pandemics to support child safety, academic development and wellbeing.

## Introduction

**Fig 1:**
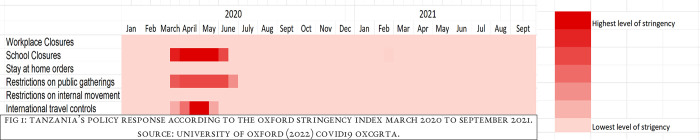
Tanzania’s policy response according to the Oxford Stringency Index March 2020 to September 2021.

School closures are a common policy response to viral epidemics [1]. The first COVID-19 case in Tanzania was detected on March 15^th^, 2020, in Arusha region. The Tanzanian government did not implement a total lockdown but encouraged the public to take personal protective hygiene measures including handwashing with clean running water and soap, use of sanitizers, observe physical distancing and mask-wearing, and banned international travel [2]. The government also decided to close all schools from kindergarten to universities in all regions of the country for three months (from March to May 2020) as a way to reduce transmission [3]. The timeline is reflected in the below (Fig 1). All primary, secondary, high schools and colleges re-opened on 1^st^ June 2020 [4].

The school environment is an avenue for students to acquire academic, social and emotional skills as well as to engage in physical activity for their development and well-being [5,6]. School closures and other lockdown measures implemented during the pandemic have been shown to be associated with a variety of risks for children [7] such as vulnerability to sexual encounters, witnessing of intimate partner violence [8], child marriage and teenage pregnancy [9], and other protection concerns including orphanhood and forms of exploitation [10] Evidence demonstrated that rates of violence and insecurity increased as a consequence of the COVID-19 pandemic [11]. However, despite the increased trend of violence during the pandemic, they were invisible and little action were taken due to the restrictions posed to control the control as such there was reduced access to health, protective and legal services [7]. Pandemic response measures also affected children’s mental health [12]. Specifically, a study conducted among students in New Delhi, India demonstrated that the lockdown led to psychological issues such as frustration, stress, and depression [13]. Studies from other countries revealed similar experiences attributed to other epidemics such as Ebola in West Africa, Influenza in North Africa and beyond [14], and Swine flu and other strains of SARS. School dropouts, risk of child labour and exploitation and distance learning difficulties due to poor digital technologies were also observed [15].

In South Africa, studies found diverse impacts of lockdown measures in terms of nutrition and mental health outcomes and learning opportunities linked to teaching disruption. These effects appeared to be more pronounced among poorer families with little access to books, electronic gadgets, and internet connection [16,17]. There was poor access of students to teachers’ support, to peers and to school-based child protection resources [18]. However, comprehensive studies on the psychological or mental health, and social and economic impacts of COVID-19 measured on displaced communities are limited, especially in Sub-Saharan Africa.

Disruption to the education sector due to COVID-19 impacted family incomes and social interactions in a myriad of ways. In many settings, school closures forced parents to spend more time with their children at home and to provide teaching and learning support, to cover expenses for meals that were generally covered by the schools, and to have reduced social interactions outside the home environment [12,19]. Some positive reflections were reported such as strengthened social cohesion due to staying indoors with their families [20]. However, it is not clear to what extent these findings were applicable across settings characterised by severe resource constraints, lower access to technology and internet, and varying social norms including refugee and humanitarian contexts.

During COVID-19, it was anticipated that infection would be higher among densely populated communities such as refugee camps, leading to rapid transmission. School closures were a compulsory measure everywhere in Tanzania, including in Kigoma Region where Nyarugusu camp is located. Refugees were mandated to stay within the premises of their compounds and continue to take other preventive measures [21]. Evidence from other continents, Asia for example, has shown that the pandemics might indeed have important health consequences in refugee settings because of overcrowding and heightened resource constraints [22].

In Tanzania, the impacts of school closures on the experiences and well-being of the extensive refugee population have not been documented. This paper presents findings from a qualitative study designed to explore COVID-19 related school closures in Nyarugusu refugee camp. We aimed to describe teachers’ and students’ experiences and perceived consequences of school closures in order to inform preparedness plans for future pandemics.

## Materials and methods

### Study site and design

The study was conducted in the Nyarugusu refugee camp, located in Kigoma Region in the Western part of Tanzania. The camp was established in 1996 and since then it has been hosting refugees fleeing conflict occurring in the neighbouring countries of the Democratic Republic of Congo (DRC) and Burundi. The latest UNHCR data indicated the camp hosts around 150,000 refugees. Of these, 80,000 are Congolese while around 70,000 are Burundians [23]. In the camp, refugees rely on rations of food and basic supplies from UN agencies and main sources of income primarily rely on small scale farming and business at small internal markets or exchanges with the hosting communities that happen sporadically.

In this site, a cluster randomized control trial (RCT) of EmpaTeach, a behavioural intervention aimed at preventing violence in schools, was implemented [24]. As described in detail elsewhere [25], the RCT collected data from school teachers and students at three time points (baseline before the implementation of the intervention, midline, three months after intervention, and endline, nine months after the implementation of the intervention). The COVID-19 pandemic was reported in the country at the end of this larger study; therefore, qualitative end-line data collection was conducted after school closures had been implemented and questions about the impact of school closures and study participants’ coping mechanisms were included to the endline interview guides.

### Study population and sampling methods

A purposive sampling approach was employed to select targeted study participants from the primary and secondary schools included in the randomised trial. With the support from the head teachers, participants were recruited when the study was commencing at the baseline in January 2019 and were interviewed in all three phases as described above (midline in May-June 2019 and endline in September 2020). A total of 44 participants (29 teachers and 15 students) were recruited (Table 1) and the selection criteria included the level of violence experienced in Congolese and Burundian schools as described elsewhere [24]. We aimed to include a sample that allowed us to reach theoretical saturation, where no new themes or concepts would be emerging from the data. There was no refusal, and all participants fully participated to the interviews.

**Table 1:** Recruitment of study participants.

Types	Congolese	Burundians
Teachers	14	15
Students	8	7
**Total**	**22**	**22**

### Theoretical framework

The study used a theoretical framework rooted in phenomenology for analysis, documenting emerging findings on the experiences of teachers and students about COVID-19 restrictions and school closures. The phenomenology implies gathering information directly from those who are impacted by the phenomenon to avoid the prejudice that might have been caused by a third party. Therefore, findings emanating from questions such as ‘*how have you perceived the COVID19 measures including school closures*? *what were your experiences during implementation of school closure and movement restrictions*? *How about teaching ability*, *academic progress*, *and impact on your life*, *the family and society*? were documented. The questions reflect the two phenomenological terms that express intentionality. The first is ‘noema’, which is the objective statement of conduct or experience as actual, and the second one is ‘noesis’, which is the subjective reflection of the objective statement [26]. Both terms facilitate understanding of how teachers and students experienced COVID-19 and how they perceived the school closures and their consequences.

### Data collection procedures

In-depth interviews (IDIs) were conducted face-to-face with teachers and students from the selected schools during endline. The study themes focused mainly on their perceptions, experiences, and consequences of COVID-19 during school closures, whether there were advantages gained, how they coped with challenges or changes they might have noted, and what could be done in future should outbreaks with similar consequences occur, particularly to ensure the safety, security and wellbeing of the people living in such settings. The interview guides were reviewed collaboratively with research partners and agencies active within the refugee camp to fit the local context and relevancy. A private place for interviews was sought within the schools to ensure freedom of expression and prevent interruptions from other people. Saturation was observed so interviews stopped when no new information was coming from the interviewees. Interviewers included males and females who were familiar with Kirundi and Congolese language and resided in the camp. Interviewers had experience in conducting qualitative studies and they piloted the tools and adjusted questions before the beginning of fieldwork. They were trained on the purpose of the study and on how to conduct interviews with participants. Study participants were introduced to the study objectives and written informed consent was sought for their participation before the start of interviews. Each interview lasted between 30–45 minutes.

### Data management and analysis

All interviews were audio recorded after seeking consent from the study participants which were complemented with field notes. The interviews were conducted in Kirundi for the Burundian respondents and Congolese Swahili for Congolese respondents. Transcription was done verbatim and translated into the English by experienced and skilled researchers. Verification of the written transcripts by senior researchers was done after listening to the audio recording to confirm the correctness of the transcriptions. Codes were developed to reflect the emerging themes. The coding process was done by two coders using Excel spreadsheets. Based on the phenomenological approach, researchers identified the meaning of the phenomenon that was explored and the relationship to different segments linked to it [26]. A conventional content analysis was conducted [27] where coding categories were derived directly from the text data, after having gone through the transcripts and field notes to gain a sense of messages contained in the data. Codes developed were grouped into themes such as: “experiences of COVID-19 control measures including school closure that were implemented”, “perceived consequences of school closures” etc. Themes included sub-themes such as “increased fear, anxiety, and depression”; “deterioration in studies”; “gender-based violence among students”; and “perceptions of school reopening”, etc. Patterns of the way the responses were evolving including similarities and differences were documented during the analysis. Quotations were taken to represent the informants’ own words.

### Ethical considerations

Ethical clearance was obtained from the Medical Research Coordinating Committee of the National Institute for Medical Research (ref. NIMR/HQ/R.8a/Vol. IX/2920) and the London School of Hygiene and Tropical Medicine. Permission to collect data in Nyarugusu Refugee Camp was granted by the Ministry of Home Affairs and camp authorities. Study participants were informed of the study and written informed consent was sought before interviews. For students under 18 years, informed consent was sought from school head teacher who were considered as the official guardian during school hours, thereafter students were requested to offer written assent. Only investigators had access to participants’ information, so confidentiality, anonymity, and privacy were maintained throughout the study and participants were free to withdraw at any time and for any reason without penalty.

## Results

Among the 44 study participants who consented to be interviewed 15 were students (age range 11–20 years, 8 females and 7 males) whereas 29 were teachers both from Congolese and Burundian schools (10 females and 19 males). The findings are presented using the following themes: 1) experiences on COVID-19 control measures including school closure that were implemented in the camp; 2) perceived consequences of COVID-19 school closure including i. increased fear, anxiety, and depression, and ii. deterioration in studies, iii. experiences of gender-based violence among students; and 3) perceptions of school reopening.

### Experiences of COVID-19 control measures including school closures

The COVID-19 pandemic was generally found to be frightening by everyone interviewed in the camp. Teachers and students expressed their fear of being infected. Fear was attributed to the high risk of death associated with the disease and therefore everyone was worried about catching the virus. Both students and teachers reported to have lived in fear of either personally getting infected or that their relatives would. Some of the students interviewed expressed as follows:

*I feel bad when I hear about Coronavirus because I fear to die if I will get infected* [Student, 14 years, female, Primary school, Burundi].

When asked to identify if they knew the measures recommended for the prevention of the virus, feedback obtained was encouraging. As expected, almost all study participants were aware of protective measures, as reflected in this quote:

*“I remember that leaders told us many things such as it is strictly prohibited to shake hands and hug each other*, *the need to wash hands with running water and soap*, *maintain a social distance of about two meters between one person and another whether sitting or standing*, *avoiding touching the mouth or nose before washing your hands*. *Also*, *wearing a mask and covering the mouth when yawning to protect others* [Teacher from Burundian school].

Such statements were common throughout the interviews and these measures were perceived to be important because they lowered the risk of infection.

School closures were mentioned as an intervention to restrict movement to reduce risks of COVID-19 infection.

*“Schools were closed due to the coronavirus pandemic*, *whereby we were supposed to stay home so that we could continue protecting ourselves*. *This was because if we continued coming to schools considering the students’ population*, *we could have gotten infected by the virus*.*”* [Student, 21 years, male, Secondary school, Burundian].

The majority of study participants said that school closures went hand in hand with the banning of social gatherings.

### Perceived consequences of school closures

Few participants claimed not to have found COVID-19 an issue affecting their lives as shown by the following justification from a teacher:

*“There was no negative impact caused by the closure of schools*. *Instead*, *it was just to simplify life for students or teachers as well as society to be in harmony due to the presence of Coronavirus disease so that we can know how to prevent ourselves from it*.*”* [Teacher, primary school, Burundian].

Teachers further argued that school closure periods were the best time for parents, guardians and even siblings to take advantage of making follow-up on children’s academic homework. Some students provided their experiences during school closure activities such as helping their parents with farming activities and cutting grass for tamed animals like goats and cows, while girls assisted their mothers with domestic work such as cooking and washing. The following statements attest:

*“I spent less time playing*, *I spent more time in farming activities*, *market activities or other activities helping adults at home*.*”* [Student, 13 years, female, Primary school, Burundian].

Similar reports were offered by teachers as well. One student expressed being pleased with spending private time studying things that were not part of the formal academic curriculum. Despite these positive reflections, some negative consequences were reported as well as detailed below.

#### Increased fear, anxiety and depression among teachers and students

Study participants reported that their mental health was negatively affected, partly because of the shock of learning about the government’s closure of schools until further notice. Some of the participants believed that the decision to close schools mirrored the unpredictability of situation in the general population’s health, whereby those living in camp were perceived to be at a higher risk. This in turn led to stress and hopelessness:

*“I was shocked when the school was closed” and “I was stressed because schools were closed and many other things were closed*. *This made a person think that maybe it is the end of the world*.*”* [Teacher, secondary school, Congolese].

Like teachers, students were also reported to have been psychologically affected by increased fear, loneliness, uncertainty, anxiety, and depression due to COVID-19 restrictions. Some said as follows:

*I felt sad when schools were closed because I was no longer learning*. *I missed school*, *I felt lonely I wished we could just continue with our studies* [Student, 13 years, male, primary school, Burundian].

Some teachers further reported having lost the freedom to move around and felt that they were forced to live like prisoners.

“*People were not allowed to walk around*, *they were told to stay at home*, *we didn’t have freedom to move around*, *everyone was worried and most of the time we had to be indoors*.*”* [Teacher primary school, Congolese]

Most of the students who wanted to finish their studies as per their academic year also felt disappointed. They felt the decision held them stagnant since their studies were paused for an unknown period, causing stress for them. They testified to have lost hope for continuing with their studies. Teachers reported that school closure led some students to consider other life options including marriage.

*“…many students left the school as they were convinced to get early marriage and others get early pregnancy…”*[Teacher, primary school, Burundi]

Students and teachers reported that school closures and the associated measures of restricting unnecessary movement reduced social interactions among members of the school community.

*“People were not allowed to walk around*. *They were told to stay at home… We did not have the freedom to move around*, *so everyone was worried and most of the time we had to be indoors*.*”* [Teacher, secondary school, Congolese].

For some participants, greeting each other by shaking hands was important as it traditionally symbolizes trust and brotherhood between individuals. With COVID-19 restrictions, participants explained how difficult it was for them to deviate from their used cultural norms when they met.

*I was affected by the condition because it was not easy to meet with my friend and not shake hands considering our greeting styles in Burundi of shaking hands and hugging each other* [Teacher, primary school, Burundian].

The majority of participants stated to have observed reduced student-student interactions, hence, missing their friends, playing and studies. The above testimonies demonstrate wide acknowledgement that COVID-19 pandemic preventive measures were well-intended but that they had negatively impacted interpersonal relationships. Most teachers also reported to have been stressed because of staying idle without using their professionalism.

*I became overstressed during the school closure which led to sleepless nights* [Teacher, Secondary school, Burundian].

Teachers showed concerns about their teaching programme having been interrupted, leading to discontinuity in the teaching syllabus. They worried whether students would be allowed to advance to the next class. They also feared their final examinations may be pushed back. In support, a teacher from Congolese school commented:

*“Closure of school because of coronavirus has affected my work because when we start the year*, *we have to prepare what to teach but when we got to the part [of COVID-19]*, *I did not finish the things I had prepared*… *we were interrupted*. *When we came back after three months*, *we did not have time to finish the programs we had planned for kids to end the year*.*”*

Teachers said that they were stressed because of the possibility of lagging behind in the academic circle, especially in their professional career practice. They claimed that their professionalism was at risk during school closures and their teaching plans were not met:

*“It affected my job because I was not using my professionalism*. *The development of my teaching profession was affected because a teacher needs consistency*, *if you stay two*, *three to four weeks out of school*, *you start forgetting even what you already know*.*”* [Teacher, Primary school, Burundian].

Other teachers complained of having an unsettled mind due to being unsure if assignments given to students could be completed during closures as they were used to be monitored by their teachers. It was difficult to enforce homework completion and practice among students when they were outside class or school compounds. Teachers further worried about the fact that outside school compounds students might be exposed to protection risks, o especially in the absence of close supervision from their parents.

#### Deterioration on study progress in schools

Study participants reported discontinuing their studies due to interruption of classes. The pause in the curricula made teachers worry that students would lag behind.

*“A student in the academic year has a certain plan…like this year I will finish this class level*. *He expected every day to get lessons prepared by his teacher*, *but it happened suddenly and they were not prepared*. *They were told ‘We stop according to time*.” [Teacher, Secondary school, Congolese].

Students’ engagement in domestic and family work appeared to limit time for self-study.

*“I spent less time studying because most of the time I was doing domestic work*.*”* [A student, 16 years, female, Primary school, Congolese].

A widespread lack of school preparedness for distance teaching and lack of internet connection limited learning activities during closures., Students reported spending their time engaging in non-academic related activities such as playing with fellow children, friends, and peers around the area.

Some students also reported leaving their homes to spend time on the river with the excuse of going to wash their clothes etc. One student viewed her engagement in domestic and family work as being a good thing, but it minimized time for private studying at home due to being too occupied and exhausted, making it hard to concentrate. Only a few acknowledged receiving supports from their family.

Lack of learning support through direct supervision from teachers during school closures was also reported to have held back students’ academic progress:

*“I did not get any support from teachers when we were not going to school*.*”* [Student, 20 years female, Secondary, Burundian].

Students’ reports were substantiated by teachers who also confirmed that no learning support was given to students during school closures. Prohibitions of social gatherings and the absence of modern learning technology emerged as reasons for not providing support to students. A teacher from Burundi commented:

*“It was not possible to organize the learning support*. *We tried to find a way to do it but we failed because it was strictly prohibited to work together and we had no machines like tablets*, *computers etc*.*”*

Despite the negative impacts noted overall, school closures fostered students’ self-learning at home for most. Several students attested to having spent more time self-studying while at home, doing revisions through reading the exercise books, and internalizing and understanding notes in preparation for various examinations that were to happen after school reopening. Committed students were able to develop an independent culture of following a study timetable while away from their teachers’ supervision. A student commented:

*“I spent night hours revising what I have been taught*, *this has helped me so much after going back to school since we were supposed to do examinations when we opened school*.*”* [Student, 27 years, male, Secondary School, Burundi].

Teachers were able to teach class six students through the formation of groups who were later given homework to keep them busy and engaged with school activities. Others were just given some instructions and questions/problems to solve.

*“Teachers prepared questions and distributed them to their homes to be done by students during COVID-19 time*.*”* [Teacher, secondary, Burundian].

Some teachers offered advice to students they encountered in the streets by chance and gave them homework to do through neighbours who lived close to them.

*"By the time schools were closed teachers and school leaders took the initiative to help students by sending them homework to do while at home*. *Therefore*, *teachers- we put efforts to make sure that they continue revising while they are still at home*.*"* [Teacher-Primary School, Burundian]

Modern technology was mentioned as one of the facilities that was lacking in the camp that would have helped people cope with the unavoidable changes caused by COVID-19.

*“We were not allowed to stay in groups and the school was not prepared to continue teaching students during school closure…We had no internet*, *which is why we failed to assist students to learn when schools were closed*.*”* [Teacher, Primary school, Congolese].

### Exposure to gender-based violence among students

School closures were widely perceived to have contributed to gender-based violence. Interviewees reported that girls became vulnerable to unwanted pregnancies due to engagement in premarital sex or the decision to enter early marriages. Teachers raised such concerns. Some relayed stories of girls who were raped in the course of interacting with men or boys due to lack of parents’ or guardians’ supervision. Pregnant girls were also suspected of possibly being infected with HIV and other sexually transmitted diseases due to having unprotected sex.

*“Many students became pregnant*, *others dropped out of school during the re-opening*, *others might have contracted HIV through sexual intercourse while others became thieves*. *Others gave up as they lost hope for the future and thought only about death*.*”* [Teacher, Burundian refugee camp side].

Probed further about how girls got pregnant during school closures, one of the reasons given by the majority of teachers was lack of parental supervision at home as testified below.

*“Students were just roaming around the streets and also lacked parental guidance as schools were closed*.*”* [Teacher, primacy school Burundian]

School closures were widely discussed as having led female students spend most of their time wandering and meeting with untrustworthy men who lured them with gifts into forms of transactional sex. “*They were roaming around*, *there were many accidents to children*, *but parents were unable to control them*, *and there was wastage of time as they were not studying*.*”* [Teacher, Primary school Congolese].

Teachers and students gave testimony regarding their knowledge of some female students who were not sleeping at home, a factor contributing to early marriage and pregnancy.

*“Students practiced bad behaviours like sleeping outside their homes which resulted into both boys and girls getting married*.*”* [Teacher, Primary school, Burundian]

Teachers said that neighbours, tax, and truck drivers were the ones pressuring female students into marriages instead of supporting their parents to protect them.

*“Other female students like some of those who are in standard nine got married after being lured by their neighbours that they are mature enough and that there is no need for more schooling*.*”* [Teacher, Primary School, Burundians].

Participants said that not everyone was sympathetic or compassionate with girls who got pregnant. Some of the study participants perceived pregnancy as the result of students’ carelessness or disobedience to the advice and guidelines provided by parents at home and teachers at school. One of the teachers said that once female students got pregnant, they were stigmatized for the lack of discipline and so no help was provided to them.

*“No any help that can be given to a student who faces challenges like pregnancy because after getting pregnant or misbehaving*, *she goes back home and because she did not wait to complete her studies and threw herself on that path*, *she gets no help*.*”* [Teacher Primary School Burundian].

Given the negative impacts of COVID-19 on children during school closures, study participants were asked about the involvement of child protection systems during the pandemic. Almost all participants acknowledged weaker protection protocols, and this contributed to increased vulnerabilities for students as there was no help and protection provided by formal systems for children generally and especially for girls who got pregnant, hence they were left at home unattended.

### Perception of school re-opening

The majority of the students interviewed felt that returning to school was an awaited opportunity and children were waiting for the government to make an announcement. Some found that staying at home affected their academic and life goals, so they were praying for immediate school re-opening. Meeting friends, and teachers and continuing with studies was the main driver of the happiness of being back in school. In a statement similar to what others gave, one Burundian secondary school student commented: *“I was very happy to return to school*, *I missed my friends*, *teachers and studying”*.

Despite their general enthusiasm about attending school after re-opening, few teachers were surprised to learn that there were drop-outs. Some girls did not show up and during follow-up checks, it was found out that they were married or pregnant. Teachers called for the intervention of child protection agencies in supporting school re-entry for those who had dropped out.

*“Regarding the pupils who encountered difficulties during the closure of schools*, *I can only suggest that [organisations] make an effort to locate them and inquire about the specific issues they encountered from each of them*. *This is because other students became pregnant while residing at the houses of their mothers and fathers*. *Some of them got married and divorced two days later and were left wondering whether they should return to their husbands or their parents*. *Thus*, *at this moment*, *these organisations can conduct investigations through teachers and guardians like us*, *enabling us to provide them with comprehensive data regarding any pupil who has had such difficulties*.*”* [Teacher, Primary school, Burundian].

Poor performance was reported and was attributed to negligence and laziness during school closures.

*“Many pupils did not achieve their full potential in the classroom*. *Exam results showed that a few students had fallen from first to tenth place*. *This resulted from the fact that during the Coronavirus*, *many students engaged with uneducated youngsters on the streets*, *spending more time there than studying*.*”* **[**Teacher, Primary school, Burundian].

Teachers had to put in more effort to help pupils who displayed deficiencies. They expressed dissatisfaction at the high rates of students forgetting what they had previously learned. Teachers had to reintroduce lessons that had been covered in the previous year because students had forgotten.

## Discussion

During COVID19, school closure was implemented worldwide as an approach to cut down transmission of Corona Virus. However, various concerns were raised. In this study, the findings revealed that school closures brought negative consequences to teachers and students in Nyarugusu Refugee Camp. The restriction to movement brought fear, worries and stress. Communities found it unusual, as they were used to interacting with handshaking and physical contact. People felt that it was difficult to predict when normal life would resume and felt stressed about risk of illness and death. Similar experiences were reported during other health crises such as during the Ebola outbreak [15]. Furthermore, the limited availability of digital technology concerns about students’ ability to complete their studies caused teachers to worry about how the curriculum might be resumed after the reopening of schools, similarly to other contexts [28].

School closures were generally perceived to have increased existing inequalities and protection risks for children, especially for those with special needs [29]. Our findings support this by showing that teachers and students reported concerns around the protection and health consequences of school closures. Increased vulnerability to unwanted pregnancies, early marriages, transactional sex, and engagement in harmful behaviours was attributed to lack of supervision from parents.

Reports from several national and international organisations show that gender-based violence and other protection concerns are widespread in Tanzania, including in refugee settings [30]. Concerns existed around the possibility that school closures and other COVID-19 response measures would exacerbate the situation(29). Our study findings indicate that the negative impact of the school closures related to increased violence and protection risks for students were substantiated by teachers in Nyarugusu Refugee Camp. These findings suggest that adults in this setting attributed increased risks due to the lack of close monitoring or supervision, in line with evidence from other non-humanitarian settings in Tanzania [31] and other contexts [32,33]. In other countries, school closure were found to have intensified the food shortage crisis and also the risk of sexual abuse due to changes in households living composition [29]. The protection risk and protective factors for children are affected by a myriad of factors and it’s important to establish monitoring systems that assess whether traditional or existing prevention and response mechanisms are effectives at times of great change at all levels of the socio-ecological model such as in the context of global pandemics.

### The role of parents and community

This study’s disturbing finding is that students faced stigma after becoming pregnant and were held accountable for their actions, with little sympathy from the community at large. Important issues still need to be answered, such as why these girls are being held responsible. How do the family and community power structures operate? This necessitates developing systems for nurturing and safeguarding children and adolescents both in and out of schools, as their young age makes them particularly susceptible to abuse and other forms of violence. It’s crucial to remember that their exposure and age prevent them from making informed decisions in settings and circumstances where abusers may easily take advantage of them. Multisectoral coordination including prioritizing primary prevention of violence and empowering children and adolescents in decision making has been recommended elsewhere [7].

The study’s findings regarding teenage pregnancies and early marriage serve as evidence of the need for closer parental supervision of children to shield them from sexual abusers and other violent offenders, which can have both short-term and long-term effects on the health and well-being of the children in the household. It is important, therefore, for parents to always cooperate with the authorities responsible for child protection, by paying greater or special attention to those at most risk such as girls and the youngest ones, especially in refugee settings because of the violence history of refugees which might change their moral ethics to becoming perpetrators of violence [34].

### Academic disruptions

School closures have taught us valuable lessons about the advancement of digital technology in education, particularly in terms of easier access to resources that support online learning. This is demonstrated by this study, which has indicated that teachers struggled to keep up with the syllabuses and with monitoring of students’ academic progress. Similar challenges have been observed in other contexts [12,35], including neighbouring countries of Uganda, Kenya, Rwanda, Burundi, and DR Congo. The lack of digital technology and little follow-up at home made students lag behind academically as they engaged in non-academic activities. Implications were observed at school reopening as teachers had to use extra time and energy to assist students as they were approaching the national examinations, a challenge that was also reported elsewhere [21]. Similar experiences were reported in South Africa where students were observed to have lower academic performance [36]. Such experiences suggest a call for the adoption of distance learning programmes to support even the refugees living in camp settings. School dropout was also observed during the reopening, confirming what was earlier reported in the ‘flash appeal for COVID-19’ that in Tanzania even before the COVID-19 pandemic, a good number of children were out of school, following the school closure intervention, more school dropouts likely to be after schools were re-opened.

### Mental health challenges

In this study some participants perceived the presence of COVID-19 as the end of the world, and there was anxiety and confusion among both teachers and students in the camp about what would happen next. Similar experiences were also reported in other studies where mental health effects including child stress, sadness, frustration and indiscipline due to loneliness, inability to performing daily activities such as teaching and attending school for students were recorded [12,16,28]. A study by Human Rights Watch documented the same challenges, where many students shared feelings of stress, anxiety, isolation, and depression associated with a lack contact with teachers and fellow students [29] Self-studying was perceived to be stressful due to the inability to access counsellors who were in school as reported in Kenya in the same study. Likewise, caregivers from DRC also reported a lack of emotional and social support from schools.

### Lesson learnt

It is important to note that schools have the important function of facilitating proper time management and mentoring of students. However, during the COVID-19 time, this was greatly impaired, and at re-opening, dropouts were attributed to early pregnancies and marriages were noted as also reported in the Human Rights Watch report. It is time for the child protection committees and other institutional and community-based structures across setting to include child-centred emergency and disaster preparedness guidelines and plans to support families and communities and be held accountable for the welfare of the children. such that each community member will perceive ‘**the neighbour’s child as belonging to him/her**." This will prevent to a larger extent the actions done by the perpetrators intentionally where sometimes are close relatives and it will also change blaming of children for the observed impacts.

## Strengths and limitations of the study

The study had several strengths. First, it provides rare insights into the experiences of refugee teachers and children during the COVID-19 pandemic. The study was conducted soon after school reopening took place in Tanzania, making it possible for the study participants to clearly recall their experiences. Our sample included a mix of teachers and students, offering a variety of different perspectives about the impacts of school closure in this setting, contributing to a richness of information and reducing bias. The qualitative approach enabled the gathering of detailed information that would have been difficult to capture with quantitative methods. Relying on research assistants from the camp setting who were familiar with local languages enhanced the accuracy of the information collected. Our study also had some limitations. Since it was conducted in the context of a broader study, our interviews could only include few additional questions about teachers’ and students ‘experiences of the pandemic therefore limiting the amount of information we could collect. Additionally, since our study was school based we were unable to gather perspectives from a wider range of stakeholders such as parents and community members which did not allow us to triangulate some of the statements presented here. Moreover, since the study involved only a small sample of teachers and students from a specific refugee setting, the information cannot be generalized to other settings given the variety of response measures implemented during the pandemic across different countries and settings.

## Conclusion

The study findings have shown that fear, worry, and anxiety affected both teachers and students as a result of COVID-19 and school closures. Teachers found the three-month school closure challenging since they were unable to rehearse their lessons due to poor access to digital technology that prevented them to implement online learning. Engaging in extracurricular activities caused students to lag behind academically and made them more susceptible to protection risks. These consequences demonstrate the need for the governments and formal and informal protection systems to prepare and plan for the mitigation of possible social, economic, and psychological impacts caused by pandemics. Activating child protection committees and other informal protection structures, enforcing access to and use of digital technology in schools, and providing support to parents and caregivers are crucial for improving the continuity of education while students are at home and preventing future protection risks and other harms.

## Supporting information

S1 Checklist COREQ (COnsolidated criteria for REporting Qualitative research) checklist. (DOCX)

S1 File Interview guides. (DOCX)
