# Supplementary material for: Experiences of teachers and students on school closures and its consequences during the COVID-19 pandemic in Nyarugusu refugee camp, Tanzania
Source: PLOS Glob Public Health. 2024 Mar 18;4(3):e0002917. doi: 10.1371/journal.pgph.0002917 (PMC10947662; doi:10.1371/journal.pgph.0002917)
Supplement: S1 Checklist — (DOCX) [file pgph.0002917.s001.docx]

**COREQ (COnsolidated criteria for REporting Qualitative research) Checklist SHAYO, EH**

| **Topic** | **Item No.** | **Guide Questions/Description** | **Reported on Page No.** |
| --- | --- | --- | --- |
| **Domain 1: Research team** | | | |
| **and reﬂexivity** | | | |
| *Personal characteristics* | | | |
| Interviewer/facilitator | 1 | Which author/s conducted the interview or focus group? | 7 |
|  |  |  |  |
| Credentials | 2 | What were the researcher’s credentials? E.g. PhD, MD | 7 |
|  |  |  |  |
| Occupation | 3 | What was their occupation at the time of the study? | 7 |
| Gender | 4 | Was the researcher male or female? | 7 |
| Experience and training | 5 | What experience or training did the researcher have? | 7 |
| *Relationship with* | | | |
| *Participants* | | | |
| Relationship established | 6 | Was a relationship established prior to study commencement? | 7 |
|  |  |  |  |
| Participant knowledge of the interviewer | 7 | What did the participants know about the researcher? e.g. personal goals, reasons for doing the research |  |
|  |  |  | 7 |
| Interviewer characteristics | 8 | What characteristics were reported about the inter viewer/facilitator?  e.g. Bias, assumptions, reasons and interests in the research topic | NA |
|  |  |  |  |
| **Domain 2: Study design** | | | |
| *Theoretical framework* | | | |
| Methodological orientation and Theory | 9 | What methodological orientation was stated to underpin the study? e.g. grounded theory, discourse analysis, ethnography, phenomenology, content analysis |  |
|  |  |  | 8 |
|  |  |  |  |
| *Participant selection* | | | |
| Sampling | 10 | How were participants selected? e.g. purposive, convenience, consecutive, snowball | 6 |
|  |  |  |  |
| Method of approach | 11 | How were participants approached? e.g. face-to-face, telephone, mail, email | 6 |
|  |  |  |  |
| Sample size | 12 | How many participants were in the study? | 7 |
| Non-participation | 13 | How many people refused to participate or dropped out? Reasons? | 6 |
| *Setting* | | | |
| Setting of data collection | 14 | Where was the data collected? e.g. home, clinic, workplace | 7 |
| Presence of non- participants | 15 | Was anyone else present besides the participants and researchers? |  |
|  |  |  | 7 |
| Description of sample | 16 | What are the important characteristics of the sample? e.g. demographic data, date | 7 |
|  |  |  |  |
| *Data collection* | | | |
| Interview guide | 17 | Were questions, prompts, guides provided by the authors? Was it pilot tested? | 7 |
| Repeat interviews | 18 | Were repeat inter views carried out? If yes, how many? | NA |
| Audio/visual recording | 19 | Did the research use audio or visual recording to collect the data? | 7 |
|  |  |  |  |
| Field notes | 20 | Were ﬁeld notes made during and/or after the interview or focus group? | 7 |
| Duration | 21 | What was the duration of the inter views or focus group? | 7 |
| Data saturation | 22 | Was data saturation discussed? | 7 |
| Transcripts returned | 23 | Were transcripts returned to participants for comment and/or correction | NA |
|  |  |  |  |
| **Domain 3: analysis and findings** |  |  |  |
| \| **Data Analysis** \| 24 \| How many data coders coded the data? \| 10 \|  \|  \| \| --- \| --- \| --- \| --- \| --- \| --- \| \| Description of the coding tree \| 25 \| Did authors provide a description of the coding tree? \| 10 \| 9-10 \|  \| \| 10 \|  \| \| Derivation of themes \| 26 \| Were themes identiﬁed in advance or derived from the data? \| 10 \| 9 \|  \| \| Software \| 27 \| What software, if applicable, was used to manage the data? \| 10 \|  \| \|  \| \|  \| \| Participant checking \| 28 \| Did participants provide feedback on the ﬁndings? \| 10 \| \| \| *Reporting* \| \| \| \| 12-21 \|  \| \| Quotations presented \| 29 \| Were participant quotations presented to illustrate the themes/ﬁndings?  Was each quotation identiﬁed? e.g. participant number \|  \|  \|  \| \| 12-20 \| 12-21 \|  \| \| Data and ﬁndings consistent \| 30 \| Was there consistency between the data presented and the ﬁndings? \| 10, 12-20 \| 12-21 \|  \| \| Clarity of major themes \| 31 \| Were major themes clearly presented in the ﬁndings? \| 11-20 \| 12-21 \|  \| \| Clarity of minor themes \| 32 \| Is there a description of diverse cases or discussion of minor themes? \| 18 \|  \| \|  \|  \| |  |  |  |
| Number of data coders | 24 | How many data coders coded the data? | 8 |
| Description of the coding tree | 25 | Did authors provide a description of the coding tree? | 8 |
| Derivation of themes | 26 | Were themes identiﬁed in advance or derived from the data? | 8 |
| Software | 27 | What software, if applicable, was used to manage the data? | 8 |
| Participant checking | 28 | What software, if applicable, was used to manage the data? | 8 |
| *Reporting* | | | |
| Quotations presented | 29 | Were participant quotations presented to illustrate the themes/ﬁndings?  Was each quotation identiﬁed? e.g. participant number | 9-21 |
| Data and ﬁndings consistent | 30 | Was there consistency between the data presented and the ﬁndings? | 8, 9-21 |
| Clarity of major themes | 31 | Were major themes clearly presented in the ﬁndings? | 9-21 |
| Clarity of minor themes | 32 | Is there a description of diverse cases or discussion of minor themes? | 9-20 |
