# Supplementary material for: Experiences of teachers and students on school closures and its consequences during the COVID-19 pandemic in Nyarugusu refugee camp, Tanzania
Source: PLOS Glob Public Health. 2024 Mar 18;4(3):e0002917. doi: 10.1371/journal.pgph.0002917 (PMC10947662; doi:10.1371/journal.pgph.0002917)
Supplement: S1 File — (DOCX) [file pgph.0002917.s002.docx]

**INTERVIEW GUIDE: TEACHERS**

Recently, there has been a global pandemic of COVID-19 across the world which has also affected certain areas of Tanzania. Governments and institutions took a number of measures to prevent and reduce the spread of the virus. This resulted in closures of primary and secondary schools in Nyarugusu for a number of weeks and to the interruption of certain services within the camp. I would like to discuss with you how these measures may have affected you as a teacher, as well as your students and the overall school community.

1. Have you heard about the COVID-19 (Coronavirus) global pandemic?

PROBE: What do you know about it?

1. How has COVID-19 or the response measures implemented in the camp affected your day-to-day life, if at all?
2. Are you aware of any preventive measures that have been taken in the camp or advocated by camp authorities to protect the community from COVID19?

PROBE: If yes, which measures have been taken? How have these measures affected you, if at all?

1. What was the impact of the school closures in your life, if any?
2. Does the school closure affect the students in any way? How / what if any?

**Professionally**: How did the school closures affect your job as a headmaster/ teacher, if at all?

PROBES:

Did school closures affect your professional development, if at all?

Did school closures affect your interactions with colleagues and other members of the school community, if at all?

Did school closures affect your status/identity as a headmaster/teacher? If so, how so?

**Socially:** Has the closure of schools impacted the way you spend your time and social interactions?

PROBES:

Did you experience changes in your interactions with other members of the school community? If Yes, how so?

Did you engage in any new activity/work with your school colleagues or other members of your community while schools were closed?

**Emotionally:** How did you feel about the closure of schools?

PROBES:

Did you feel happy about it? If so, why? Did you feel nervous or stressed about it? If so, why?

How did you feel on days when you did not attend school because it was closed?

1. What do you think might be the impact of the school closure on children’s well- being?
2. How do you think they spent their time while not in school?

(Probe: what were they doing, who they were hanging out with?)

1. What do you think might be consequences (positive or negative) on their learning ability and outcomes?
2. Do you think school closures will have an impact of children’s attendance and enrolment in the coming months? If yes, in which way?
3. To what extent were you (teachers and school staff) able to support students with their learning during school closures?
4. What do you think might be child protection concerns for students resulting from the temporary closure of schools?
5. Did students have opportunities to continue with their learning activities during school closures? What kind of opportunities/activities were available to them?
6. Did you and your school colleagues provide learning support to students during school closures? IF YES: What was your contribution? Was the learning support organised at camp level or school level or was it based on individual teacher initiative?

**INTERVIEW GUIDE: STUDENTS**

I would like to ask you a few questions about the period in which schools in Nyarugusu have been closed. You may remember that until a few weeks ago (between March and July 2020) schools were closed for a period.

1. Do you know why schools closed?

**[If child mentions virus or COVID-19 or disease, then ask]:**

- - What do you know about ?
  - How do you feel about it? What makes you feel this way?

1. Generally, how did you feel in the period when schools were closed? Is there anything about that period that made you feel happy or sad?
2. When school was closed, did you miss anything about it?
   - IF YES: What did you miss the most about it? Why?
   - Probe: classmates and friends? Teachers? Learning activities and studying? Games and other activities?
   - What would you say was the thing about school that you missed the least? Why?
3. When you could not go to school because it was closed how did you spend your time during the day?
   - Did you spend more/less time playing?
   - Did you spend more/less time helping out at home with chores and housework?
   - Did you spend more/less time helping with work in the fields/market/other activities that adults in your household engage in?
   - Did you spend more/less time studying?
4. When you could not go to school because it was closed did you continue studying on your own?
   - If yes, did you do it alone or with other students? If so: With whom?
   - If yes, did any member of your household help you or support you to study?
   - If yes, did any teacher help you or support you to study?
5. Did you notice any changes in your household during the period in which school was closed?
   - Did your family members do anything different than usual (such as stopping work or working more, visiting other community members more or less, asking you to do specific tasks or activities)?
   - Do you think other children in your household were happy, sad, or the same as usual during that period?
   - Do you think adults in your household were happy, sad, or the same as usual during that period?
6. Are you back in school now?

IF YES: When you went back to school:

- - Were you excited to be back in school? Or were you happier when school was closed? Why is that?
  - Who were you most excited to see when going back to school, if anyone?
    - Probes: classmates? Any specific group of friends? Teachers? Other?

END
